# In Vivo Safety Studies With SPBN GASGAS in the Frame of Oral Vaccination of Foxes and Raccoon Dogs Against Rabies

**DOI:** 10.3389/fvets.2018.00091

**Published:** 2018-05-18

**Authors:** Steffen Ortmann, Antje Kretzschmar, Christiane Kaiser, Thomas Lindner, Conrad Freuling, Christian Kaiser, Peter Schuster, Thomas Mueller, Ad Vos

**Affiliations:** ^1^IDT Biologika GmbH, Dessau-Rosslau, Germany; ^2^Institute of Molecular Virology and Cell Biology, Friedrich-Loeffler-Institute, Greifswald, Germany

**Keywords:** rabies, SPBN GASGAS, red fox, raccoon dog, oral vaccination

## Abstract

In order to obtain Marketing Authorization for an oral rabies vaccine in the European Union, not only safety studies in the target species, red fox and raccoon dog, are required. Since baits are distributed unsupervised in the environment, specific safety studies in selected non-target species are compulsory. Furthermore, oral rabies vaccines are based on live, replication-competent viruses and thus distinct safety studies in the target species for such type of vaccines are also mandatory. Here, the results of these safety studies in target and selected non-target species for a 3rd generation oral rabies virus vaccine construct, SPBN GASGAS (Rabitec), are presented. The studies included the following species; red fox, raccoon dog, domestic dog, domestic cat, domestic pig, wild rodents. The following safety topics were investigated; overdose, repeated dose, dissemination, shedding, horizontal and vertical transmission. It was shown that SPBN GASGAS did not cause disease or any other adverse reaction in vaccinated animals and naïve contact animals. The vaccine did not disseminate within the host beyond the site of entry. No horizontal transmission was observed in wild rodents. In the target species, there was evidence that in a few cases horizontal transmission of vaccine virus could have occurred under these experimental conditions; most likely immediately after vaccine administration. The vaccine construct SPBN GASGAS meets therefore the latest revised minimal safety requirements as laid down in the European Pharmacopoeia.

## Introduction

Recently, a third generation oral rabies virus vaccine SPBN GASGAS (Rabitec) received a positive opinion from the Committee for Medicinal Products for Veterinary Use (CVMP) of the European Medicines Agency (EMA) for oral vaccination of foxes (*Vulpes vulpes*) and raccoon dogs (*Nyctereutes procyonoides*) against rabies in the European Union (EU). Although this live replication-competent rabies virus vaccine does not express a foreign gene it is considered a genetically modified organism (GMO) due to the genetic modifications realized by site-directed mutagenesis. As being classified a GMO, it must follow a centralized EU-registration procedure. Part of the safety assessment of oral rabies vaccines are studies in target – and selected non-target species as described in the monograph on rabies vaccines for foxes and raccoon dogs and general chapters on vaccines in the European Pharmacopoeia (1). For oral rabies vaccines, the following specific tests are required; a single overdose in red foxes, raccoon dogs, domestic dogs (*Canis lupus familiaris*) and domestic cats (*Felis catus*) whereby the animals are observed for 180 days. Also, it must be shown that the vaccine strain does not spread from one animal to another in wild rodent populations. More general safety requirements for vaccines are studies investigating the administration of repeated doses and the effect on reproductive performance. Additional studies for live vaccines include investigating the spread of the vaccine strain to other animals (horizontal transmission). Moreover, studies are required to determine the dissemination of the vaccine virus in the body of the host with particular attention to the sites of uptake and replication. Although the dissemination studies are only required for the target species, it was also investigated for domestic dogs and - pigs (*Sus scrofa domesticus*); the latter as surrogate for wild boars (*Sus scrofa*). Wild boars are widely distributed in EU and well known bait competitors (2). Since these animals are also considered game and their meat is used for human consumption, it was deemed necessary to investigate the dissemination of the vaccine virus in this species in detail as well. As domestic dogs live in very close association with humans a dissemination study in dogs was determined essential. Also, secretions like saliva and feces from target and non-target species that received the vaccine virus should be examined for the presence of the vaccine virus; the results of these shedding studies have been published previously (3). The results of the above-mentioned studies showed that the vaccine virus SPBN GASGAS did not induce any adverse reaction in target – and non-target species and thus meets the minimal safety requirements set by the regulatory authorities.

## Material and Methods

### Ethical statement

All animals were kept at the experimental animal facility at IDT Biologika in accordance with the prevailing guidelines and the studies were performed according to European guidelines on animal welfare, clinical endpoints, and care of the Federation of European Laboratory Animal Science Associations (FELASA). The required permits for the animal studies were obtained from the appropriate veterinary authorities in the federal state of Saxony Anhalt, Germany (Landesverwaltungsamt Sachsen – Anhalt, Referat Verbraucherschutz, Veterinärangelegenheiten) (See Table S1 for approval numbers).

### Vaccine virus

The vaccine construct SPBN GASGAS is derived from SAD L16, a cDNA clone of the oral rabies lyssavirus (RABV) vaccine strain SAD B19 (4). SPBN GASGAS lacks the pseudogene (ᴪ) and has been genetically modified by site-directed mutagenesis. To abolish residual pathogenicity and reduce the risk of the emergence of a less attenuated revertant by back mutation, mutations have been incorporated at multiple sites; amino acid positions 194 (AAT [Asn] → TCC [Ser]) and 333 (AGA [Arg] → GAG [Glu]) of the glycoprotein (5). Furthermore, the construct contains a second identical glycoprotein gene with modifications as described (6, 7). The vaccine virus was prepared according to Vos et al (8). Material for the overdose studies was concentrated via tangential flow filtration using ultrafiltration flat sheet cassettes with a Molecular Weight Cut Off (MWCO) of 300 kDa.

### Assays

For detection of rabies virus in the brain, samples are tested by the Fluorescent Antibody Test (FAT) (9). To detect replication competent vaccine virus in tissues samples other than brain material the Rabies Tissue Culture Infection Test (RTCIT) was used (10). As this test involves 3 serial passages in cell culture until a sample is considered negative, samples were first screened by real-time RT-PCR for the presence of viral RNA as described by Hofmann et al (11). If positive in real-time RT-PCR, the sample was subsequently tested for infectious virus by RTCIT. To detect and quantify antibodies against rabies virus in vaccinated and contact animals, three different assays were used. For the detection of VNA, the Rapid Fluorescence Focus Infection Test (RFFIT) (12) with the modifications as described by Cox & Schneider (13) and the Fluorescent Antibody Viral Neutralization (FAVN) (14) were used. For the detection of binding antibodies against rabies virus a blocking ELISA was used according to the instructions of the manufacturer (BioPro, Rabies ELISA, BioPro, Prague,Czech Republic) (15). Primarily, the RFFIT was used but if the obtained results were inconclusive due to for example cell toxicity or unspecific reactions, a retention sample was tested by FAVN and/or ELISA. Initially, the FAVN-assay was used for these retention samples, but later replaced by the ELISA as this assay was better suited for qualifying samples unambiguous seropositive or seronegative (15).

### Animals

General information on the different animal studies can be found in Table 1 and Table S1. The vaccine was administered by direct oral instillation, except for the study in pregnant vixens. Blood samples were collected and examined for the presence of virus neutralizing antibodies (VNA) prior to vaccine administration, at the end of the study and sometimes on different occasions during the observation period of all animals, except rodents (Table 1). In case animals were sedated for vaccine administration or sampling, a mixture of Xylazine and Ketamine was administered i.m.; for the domestic pigs, methadone was used instead of ketamine. The dosage administered was adjusted to body weight. Euthanasia was performed by an intracardial injection of Release® (300 mg/ml Pentobarbital-natrium) per sedated animal.

**Table 1:** Overview table of animal studies performed (except for reproduction study all animals received material by direct oral instillation).

**Animal species**	**Study purpose**	**Number of animals**	**Volume (ml)**	**Dose (FFU/ml)**	**Blood sampling (days post vacc.)**	**ObservationPeriod (d)**
Red fox	Overdose	26	1.0	9.1	0, 42, 183	183
Raccoon dog	Overdose	26	2.0	9.1	0, 42, 182	182
Domestic dog	Overdose & Horizontal transmission	12 (+4)	1.0	9.1	0, 16, 28, 55, 90, 122, 181	181
Domestic cat	Overdose & Horizontal transmission	12 (+4)	1.0	9.1	0, 14, 28, 56, 91, 126, 182	182
Red fox	Repeated dose (0, 3 & 7d) & Horizontal transmission	8 (+4*)	1.0	7.5	0, 28	28
Raccoon dog	Repeated dose (0, 3 & 7d) & Horizontal transmission	8 (+4)	1.0	7.5	0, 31	31
Red fox	Reproduction (vertical transmission)	3 vixens, 14 cubs	1.7	7.2	week 2, 4, 8 and 12 post parturition	87 (cubs), 126 (vixens)
Red fox	Dissemination & Horizontal transmission	12 (+ 2)	1.7	7.5	0,1,2,3,5,7,10(84)	1,2,3,5,7,10(84)
Raccoon dog	Dissemination & Horizontal transmission	12 (+ 2)	1.7	7.5	0,1,2,3,5,7,10(85)	1,2,3,5,7,10(85)
Domestic dog	Dissemination	12	3.5	7.9	0,1,2,3,5,7,10	1,2,3,5,7,10
Domestic pig	Dissemination & Horizontal transmission	10 (+ 2)	1.7	7.5	0,28,56	1,2,4,8,56 (56)
Field mouse	Horizontal transmission	30 (+ 24)	0.03	9.1	n.a.	87
House mouse	Horizontal transmission	20 (+ 32)	0.03	9.1	n.a.	90
Guinea pig	Horizontal transmission	5 (+ 4)	0.5	9.1	n.a.	90

*one naïve contact fox was accidentally given a vaccine dose on the day that the third dose was administered to the vaccinated animals

Study purpose refers to the required safety test as described in the European Pharmacopoeia and the numbers of animals indicates the number of treated animals and the number in parentheses refer to the number of naïve contact animals included.

### Overdose (red fox, raccoon dog, domestic cat, domestic dog)

For the overdose studies in the target and non-target species, as required at least 10x the maximum dose most likely to be contained in a vaccine bait was administered and the animals were observed for at least 180 days (1). At the end of the study, brain tissue (pooled samples of the medulla oblongata, cerebrum [near location of cornu ammonis], cerebellum) of all animals were tested for the presence of vaccine virus using the FAT. Additionally, the gonads of the male raccoon dogs were tested for the presence of vaccine virus RNA by using real time RT-PCR.

### Dissemination (red fox, raccoon dog, domestic dog, domestic pig)

Twelve animals of each target species received 1.7 ml SPBN GASGAS (10^7.5^ FFU/ml) by direct oral instillation. Furthermore, 2 naïve contact animals of each target species were housed with one or more treated animals. On 1, 2, 3, 5, 7 and 10 days post vaccine administration 2 foxes or 2 raccoon dogs were euthanized and the following tissue and organ samples were taken and examined for the presence of the vaccine virus; brain (Cornu ammonis, Medulla oblongata, Cerebellum, and Cerebrum), Lnn. mandibularis and – retropharyngeales [only foxes], upper and lower mucosa of the oral cavity, tongue, velum palatinum, tonsils (Tonsila sublingualis, T. lingualis, T. palatine, T. pharyngica), lung, kidney, bladder, heart, colon, ileum, and jejunum. The two contact foxes and raccoon dogs were sacrificed at day 84 and 85, respectively. Twelve dogs received 3.5 ml SPBN GASGAS (10^7.9^ FFU/ml) and the same sampling scheme as for the target species was used. For the domestic pigs, a different sampling schedule was applied; on day 1, 2, 4, 8 and 56 post vaccine administration two animals were euthanized. Besides brain samples the following organs/tissues were sampled; tongue, lung, kidney, liver, heart, bladder, jejunal lymph nodes, colon lymph nodes, colon, jejunum, ileum, Tonsilla pharyngea, Lnn. mediastinales, and Lnn. tracheobronchales. Furthermore, several samples were taken from tissues that are used for meat products; venter fat tissue, venter muscle tissue, femoral fat tissue, femoral muscle tissue, Crus fat tissue, and Crus muscle tissue. Also, on day 56 both contact pigs were sacrificed, and besides a blood sample also brain tissue was collected and examined for the presence of vaccine virus.

### Repeated dose (red fox, raccoon dog)

Twelve juvenile foxes and raccoon dogs were housed in 4 groups of 3 animals each and observed for 28 and 31 days, respectively. At day 0, 3 and 7, two animals in each group received 1.0 ml SPBN GASGAS (10^7.5^ FFU/ml) by direct oral instillation. The remaining third animal in each group did not receive the vaccine construct and was kept as a naïve contact animal to determine possible horizontal transmission.

### Reproduction

Three, pregnant foxes were offered a vaccine bait containing a blister filled with 1.7 ml SPBN GASGAS (10^7.2^ FFU/ml) 8–12 days after mating. Two vixens refused the baits and were subsequently offered the vaccine by placing it in a drinking bowl. One vixen drank 14 ml and the other animal 17 ml of the vaccine construct (10^7.2^ FFU/ml). As blood sampling would require anesthesia and these activities could lead to abortion, no blood sample was taken prior to vaccine administration. However, all three animals had not been vaccinated against rabies prior to study begin. The vixens were housed in individual cages. To examine if the vaccine virus was transferred from the vaccinated vixens to their offspring, blood and saliva were collected from the cubs in week 2, 4, 7 and 12 post parturition. The vixens and cubs were observed for 83 and 87 days post parturition, respectively. At the end of the observation period all animals were euthanized and subsequently brain and salivary glands were examined for the presence of the vaccine virus.

### Horizontal transmission wild rodents (field mouse, house mouse, guinea pigs)

Both common vole (*Microtus arvalis*) and house mice (*Mus musculus*) received 0.03 ml SPBN GASGAS (10 ^9.1^ FFU/ml) by direct oral instillation and were placed in cages together with at least one naïve contact animal. As only male guinea pigs (*Cavia porcellus*) were available, the animals could not be housed together and were kept individually; hence no direct contact between treated (0.5 ml SPBN GASGAS [10 ^9.1^ FFU/ml], d.o.a.) and control animals was possible. However, the animals were kept in the same room and the nest material from the vaccinated animals was mixed every day with the nest material in the cages of the control animals. For the application of the vaccine the unsedated animals were fixed at the neck and kept nearly in vertical position; this straightened the oesophagus, allowing an easier passage of the substance. The fixation did not affect the swallowing reflex of the rodents. After fixation the pipette dip was placed 2–3 mm deep into the mouth and the substance was administered slowly. The naïve contact house and field mice were ear-punched for identification purposes.

## Results

### Overdose

All animals tested sero-negative prior to vaccine administration. After administration all foxes and raccoon dogs developed an immune response (>0.5 IU/ml), indicating successful vaccine uptake. One dog and one cat did not develop a detectable immune response in any of the samples collected post vaccine administration. Also, the 4 naïve contact cats and dogs remained sero-negative during the observation period, indicating no horizontal transmission of the vaccine virus. During the observation period, it was necessary to exchange dogs between cages to avoid injuries as a result of aggressive behaviour. The contact animals though were always kept in a cage with at least one treated animal. All vaccinated and contact animals survived the 6 months observation period, except for two vaccinated raccoon dogs that were found dead on day 107 and 108 post vaccine administration. One animal died of bacterial pneumonia with formation of lung abscesses and the other showed leucocytosis, inflammation-related lung changes and bacteraemia. Interdigital abscesses were suspected to be the cause of a bacterial sepsis which resulted in the sudden death of the animals. Furthermore, several foxes and raccoon dogs showed symptoms of impaired health for short periods during the observation period. These symptoms were unrelated to rabies and all animals recovered. Furthermore, no rabies virus was detectable in the brains of these and all other animals at the end of the study (FAT). Also, no viral RNA was detected in the gonads of the raccoon dogs at the end of the study.

### Repeated dose

None of the foxes and raccoon dog had rabies virus antibodies prior to vaccine administration. All but one fox remained healthy during the 28 day observation period: One fox was euthanized at day 23 post first vaccine administration due to an injured front leg. No RABV antigen was detected in the brains of the vaccinated and contact animals (FAT). Unfortunately, one contact fox accidentally received a vaccine dose on the day that the 3rd dose was administered; thus, the animal cannot be considered a naïve contact animal. All animals that received the vaccine had seroconverted at the end of the study, including the contact animal that was given mistakenly a single dose on day 7. Also, 2 of 3 contact foxes seroconverted (2.67 and 0.64 IU/ml) and one contact raccoon dog had VNA levels > 0.5 IU/ml (0.86 IU/ml), indicating contact with the vaccine.

### Dissemination

All foxes, raccoon dogs, dogs and pigs tested sero-negative prior to vaccine administration. Viral RNA was detected in the palatine tonsils in all foxes that received the vaccine construct (PCR), except for one fox euthanized on day 1. Also, in 9 of the 12 palatine tonsils collected from the raccoon dogs, viral RNA was detected: 3 palatine tonsils collected from animals euthanized at day 2, 5 and 7 post vaccine administration tested negative. However, viable virus was re-isolated from the PCR-positive palatine tonsils only during the first 3 days. From day 5 onwards, these samples tested negative in RTCIT indicating that no viable virus was present anymore. In dogs, viral RNA was detected in 6 palatine tonsils, but only in a sample taken from a dog on day 3 post vaccine administration viable virus was isolated (Table 2). In another dog, sampled on day 5 post vaccine administration, besides the palatine tonsil also the retropharyngeal lymph nodes and pharyngeal tonsils tested PCR-positive and subsequently RTCIT-negative.

**Table 2:** presence of viral RNA (PCR) and infectious virus (RTCIT) in the palatine tonsils of red foxes, raccoon dogs and dogs (green, PCR-negative; red, PCR-positive; +, RTCIT positive; -, RTCIT-negative).

	Days post vaccine administration											
	1		2		3		5		7		10	
Animal	1	2	3	4	5	6	7	8	9	10	11	12
Red fox		**+**	**+**	**+**	**+**	**+**	-	-	-	-	-	-
Raccoon dog	**+**	**+**	**+**		**+**	**+**	-		-		-	-
Dog		-			-	**+**	-	-				-

All other tissues and organ samples from foxes tested negative for RABV RNA, irrespective of the day of sampling. In raccoon dogs, 3 additional samples tested positive for viral RNA; both velum palatinum samples and a lung tissue sample collected on day 3 and 7, respectively. Only, in one velum palatinum sample infectious virus was re-isolated. In the domestic pigs, all samples tested PCR-negative although no sample of the Tonsilla pharyngea was collected from the animals sacrificed on day 1 and 2 post vaccine administration. No virus antigen was detected in the brain samples collected from all vaccinated and contact animals (FAT).

Only the 4 foxes sampled on day 7 and 10 post vaccine administration seroconverted (>0.5 IU/ml), the foxes euthanized earlier did not yet develop a detectable immune response. A similar situation was observed in the raccoon dogs. Both raccoon dogs euthanized at day 10 post vaccine administration seroconverted; 1.77 and 1.57 IU/ml. All blood samples collected from the other vaccinated animals were below the threshold of 0.5 IU/ml, although one sample taken from a raccoon dog on day 7 post vaccine administration was very close to this threshold (0.49 IU/ml). The result of the ELISA confirmed this by giving a positive signal (>40% inhibition). Only one dog euthanized 10 days post vaccine administration seroconverted (8.18 IU/ml). Both pigs euthanized 56 days post vaccine administration seroconverted. All contact animals from the different species did not develop an immune response. Also, no viral RNA was detected in the samples collected from these contact animals, indicating that horizontal transmission of the vaccine virus did occur during these studies.

### Reproductive performance

The 3 vixens gave birth to 14 cubs on day 39 and 43 post vaccine administration; two litters of 5 cubs and one litter with 4 cubs. The cubs showed evidence of decreasing low levels of maternally derived antibodies at day 15 (<0.4 IU/ml) and 87 after birth (<0.1 IU/ml) (Table 3). The level of antibodies in the vixens was at all sampling points > 0.5 IU/ml. In none of the cubs vaccine virus was found in saliva, salivary glands or brain. No adverse or critical event (such as abortions) referring to general health was recorded in the vixens and their cubs.

**Table 3:** Serology results (IU/ml) of the blood samples taken from the foxes, vixens and their cubs (RFFIT); the vixen was vaccinated by the oral route during early pregnancy ( -, sample not collected).

		**sample (weeks post parturition)**			
	**sex**	**B1 (2 wk)**	**B2 (4 wk)**	**B3 (7 wk)**	**B4 (12 wk)**
**Vixen 1**	F	1.03	1.29	0.95	1.12
**cub 1**	M	0.18	0.08	0.06	0.08
**Cub 2**	M	0.07	0.08	0.06	0.09
**Cub 3**	M	0.13	0.06	0.08	0.09
**Cub 4**	M	0.16	0.06	0.05	0.04
**Vixen 2**	F	3.13	3.25	2.78	4.45
**Cub 5**	M	0.27	0.15	0.10	0.09
**Cub 6**	F	0.27	0.16	0.10	0.08
**Cub 7**	M	0.36	0.20	0.08	0.07
**Cub 8**	M	0.35	0.13	0.05	0.08
**Cub 9**	F	-	0.10	0.06	0.08
**Vixen 3**	F	0.59	2.61	1.71	1.21
**Cub 10**	M	-	0.15	0.03	0.09
**Cub 11**	F	0.40	0.16	0.06	0.05
**Cub 12**	F	0.32	0.15	0.03	0.08
**Cub 13**	F	-	0.09	0.07	0.10
**Cub 14**	F	0.23	0.13	0.07	0.08
**Mean cubs**		0.25	0.12	0.06	0.08

### Wild rodents

In house mice and guinea pigs, no adverse reaction was observed in any of the animals during the entire observation period and subsequently no virus antigen was detected in the brain of the control animals (FAT). Seven common voles died during the observation period, but none of these animals showed any clinical signs of rabies as confirmed by negative FAT; 3 treated and 4 contact animals. Also, no vaccine virus was detected in the brains of the remaining contact voles at the end of the study (FAT).

## Discussion

Distribution of oral rabies vaccine baits in the environment targeted at red foxes and raccoon dogs automatically implicates that also other animal species can locate and consume vaccine baits. Furthermore, also humans can have direct or indirect contact with the vaccine in the baits. Hence, safety studies are not only required in target species as with conventional vaccines but also for non-target species. It is of course not feasible to test the vaccine virus in all non-target species present in areas where vaccine baits are to be distributed; a representative subset of potential non-target species must be selected.

Several criteria for the identification of non-target species to be tested can be used; among others, likelihood of exposure to the vaccine (incl. abundance of non-target species and palatability of bait matrix to the non-target species), susceptibility to the vaccine virus, relevance to humans (close association with humans like pets and livestock, game). Also, some practical considerations play a role; some species identified as bait competitors can be more easily tested under experimental conditions or are more likely to be available (in sufficient numbers) for such safety studies. The available oral rabies vaccines have been extensively tested in many different species (16–19). As SPBN GASGAS is a live replication competent rabies virus contained in a blister surrounded by a bait matrix, a risk assessment is restricted to non-volant mammals. Only mammals are considered to be susceptible for rabies infection, whereby bat species can also be ruled out as potential bait competitors based on their behavioral ecology. Although birds have been identified as bait competitors and experimental infections have shown that rabies virus can replicate under certain conditions in these hosts (20), birds are not considered susceptible for rabies infection under natural conditions (21). Furthermore, SPBN GASGAS is a genetically modified attenuated derivate of the widely used oral rabies virus vaccine SAD B19 and the latter was shown to be completely apathogenic in all non-target species tested, including non-human primates, with the exception of rodents (16, 22, 23). Hence, considering the outcome of these safety studies conducted with its less attenuated parental strain and avoiding redundant experimental animal studies, it was decided to restrict safety studies to the species, red fox, raccoon dog, domestic dog, domestic cat and wild rodents as required in the European Pharmacopoeia (1). The only additional species included was domestic pigs as an alternative for wild boars. Wild boars as game species have been identified as bait competitors (2). However, the risk of possible vaccine transmission to humans through consumption of meat from a wild boar that located and ate a vaccine bait is negligible as shown in the dissemination study carried out. Common voles, house mice and guinea pigs were selected for the safety studies in wild rodents, since these three species were available from institutes that kept breeding stocks of these wild rodents for research purposes. Wild rodents cannot be obtained from commercial sources and capturing wild rodents for research purposes is associated with many hurdles like obtaining (capture) permits and preventing cross-contamination with pathogens from wild-rodents to laboratory breeding stocks.

The overdose and repeated dose studies indicated that SPBN GASGAS did not induce disease or any other adverse reaction in the target and non-target species tested. Indirect evidence for horizontal transmission was found in several studies were naïve contact animals (2 foxes and 1 raccoon dog) tested positive for rabies antibodies after having direct contact with vaccinated animals. There is the possibility that this is an artifact of the housing conditions in these experiments. Similar observations were made in a control fox sharing a cage with a conspecific that was orally vaccinated with a vaccinia virus expressing the rabies glycoprotein; also here, the control animal developed rabies specific antibodies (24). However, as has been shown, vaccine virus administered can be detected in the saliva in the initial hours after oral instillation (3). Hence, animals that consumed a bait can transmit the vaccine virus to other animals (biting, social grooming) during this short time frame before it is cleared from the oral cavity; although, this is believed to be rare in the wild (24).

Although vaccine baits are generally not distributed during the gestation period of foxes and raccoon dogs in Europe (25), it cannot be completely excluded that pregnant animals can locate and consume a vaccine bait, for example during emergency vaccination campaigns (26). Hence, the vaccine was tested in pregnant animals to identify any harmful effects on the vixen and her progeny, including vertical transmission. The gestation period of the vaccinated vixens lasted between 48 and 56 days and litter size varied between 4 and 5 cubs; this all within the normal range for farmed foxes (27). Serology results indicated passive transmission of maternally derived antibodies in accordance with results obtained with the parental strain SAD B19 (28, 29). The vaccine strain did not spread to the cubs (i.e., no vertical transmission) and the oral administration of the vaccine virus is safe for pregnant animals and their offspring. Another safety issue associated with reproduction and the use of SPBN GASGAS as a GMO is the perceived risk of potential integration of viral RNA in the reproductive organs and subsequent transmission to progeny. Although SPBN GASGAS is considered a GMO it does not contain a foreign gene and the life-cycle and biology of RABV exclude genomic insertion in the DNA of the host. Therefore, the finding that no viral RNA was detected in the gonads of the raccoon dogs during the overdose study was to be expected. Actually, it was shown that the vaccine virus does not disseminate widely after uptake in the animals. It is predominantly taken up by the palatine tonsils as shown in this and other studies where after limited local replication it is cleared by the immune system (30). The presence of viral RNA in lung tissue of a raccoon dog 7 days post vaccine administration is most likely a result of administering the test item by direct oral instillation into the sedated animal whereby the vaccine entered the respiratory tract. It was shown that in comparative dissemination studies SPBN GASGAS did not have a different tropism than its parental strain SAD B19 and other oral rabies virus vaccines (16, 31).

In contrast to its parental strain SAD B19, SPBN GASGAS was innocuous when orally administered to wild rodents and also did not induce disease in contact animals, indicating no spread of the vaccine virus among rodents. The death of several treated and contact voles was not vaccine induced but most likely age-related. The normal life expectation of these mice is 10–12 months. In exceptional cases (laboratory conditions) they may also reach an age of 2–3 years. In this study the youngest and oldest mice were upon arrival 163 and 957 days old, respectively. Another required *in vivo* test in mice is showing genetic stability whereby the vaccine virus is serially passaged in suckling mice. The results of this study will be presented in a separate paper together with the genetic stability after *in vitro* passaging in the production cell line, BHK BSR Cl13, including whole genome sequencing.

## Conclusion

It can be concluded that the oral rabies virus vaccine SPBN GASGAS meets the latest revised safety requirements of the European Pharmacopoeia (1). The efficacy of this vaccine strain in the target species, red fox and raccoon dog, has been previously shown (32). Hence, this third generation oral rabies virus vaccine has retained the immunogenic properties of its parental strain SAD B19 but has a greatly improved safety profile in comparison to first and second generation oral rabies virus vaccines. The residual pathogenicity in wild rodents as observed with the parenteral strain was eliminated and the risk of reversion to virulence is negligible due to multiple genetic modifications. However, efficacy and safety of SPBN GASGAS must also be assessed under field conditions during post-campaign monitoring after distribution of vaccine baits containing SPBN GASGAS.

## Ethics Statement

All animals were kept at the experimental animal facility at IDT Biologika in accordance with the prevailing guidelines and the studies were performed according to European guidelines on animal welfare, clinical endpoints, and care of the Federation of European Laboratory Animal Science Associations (FELASA). The required permits for the animal studies were obtained from the appropriate veterinary authorities in the federal state of Saxony Anhalt, Germany (Landesverwaltungsamt Sachsen – Anhalt, Referat Verbraucherschutz, Veterinärangelegenheiten) (See Table S1 for approval numbers).

## Authors contributions

SO, AK, CK, PS and AV were responsible for study design and data analysis, CK, SO, AK, CaK (Christiane Kaiser) and TL carried out the actual animal studies and collection of samples. CF and TM were responsible for diagnostic assays. Finally, TM, CF, SO and AV wrote the article.

## Conflict of interest

SO, AK, CK, CaK, TL, PS and AV are full time employees of the company producing the oral rabies vaccine SPBN GASGAS. The remaining authors declare that the research was conducted in the absence of any commercial or financial relationships that could be constructed as a potential conflict of interest.

## Supplementary material

Table S1 Origin of animals and housing conditions (water was offered ad libitum).
