# Supplementary material for: In Vivo Safety Studies With SPBN GASGAS in the Frame of Oral Vaccination of Foxes and Raccoon Dogs Against Rabies
Source: Front Vet Sci. 2018 May 18;5:91. doi: 10.3389/fvets.2018.00091 (PMC5968751; doi:10.3389/fvets.2018.00091)
Supplement: Table S1 — Origin of animals and housing conditions (water was offered ad libitum). [file Table1.DOCX]

### Supplementary file: Origin of animals and housing conditions (water was offered ad libitum)

| Animal species | Approval number | source | Housing (cage size) | sex | Age at (first) vaccination | Feeding |
| --- | --- | --- | --- | --- | --- | --- |
| Fox (overdose) | 42502-3-658 IDT | Commercial fur farm,  Poland | Individual (3m²) | 15 ♂+ 11 ♀ | Juvenile,  122-138 days | commercial food for fur farm animals ^2^. Twice a week the animals additionally received some fruit, vegetables or adding dead chicken and mice to their ratios of standardized feed. |
| Fox (repeated dose) | 42502-3-658 IDT | Commercial fur farm,  Poland | Grouped, 3 animals per cage (>6m² + >20m²)^1^;  2 treated and 1 contact animal per cage | 5 ♂+ 7 ♀ | Juvenile,  (4-6 months) | commercial food for fur farm animals ^2^. Twice a week the foxes additionally received some fruit, vegetables or adding dead chicken and mice to their ratios of standardized feed. |
| Fox (dissemination) | 42502-3-658 IDT | Commercial fur farm,  Poland | Grouped, 2-3 animals per cage ((>6m² + >20m²)^1^;  2 cages with 2 treated and one contact animal and 4 cages with 2 treated animals | 2 ♂+ 12 ♀ | adult | commercial food for fur farm animals ^2^. Twice a week the foxes additionally received some fruit, vegetables or adding dead chicken and mice to their ratios of standardized feed. |
| Fox (reproduction) | 42502-3-658 IDT | Commercial fur farm,  Poland | Grouped, 3 cages (>6.0m² + > 20m²)^1^ with each 1 vixen and her offspring animals; | 3 ♀ + 14 cubs  (8♂ + 6♀) | Adult and new born cups | commercial food for fur farm animals ^2^. Twice a week the foxes additionally received some fruit or vegetables. After giving birth the vixens were fed *ad libitum* adding dead chicken and mice to their ratios of standardized feed. |
| Raccoon dog (overdose) | 42502-3-762 IDT | Commercial fur farm,  Poland | Individual (3m²) | 18 ♂+ 8 ♀ | Juvenile,  225-265 days | commercial food for fur farm animals ^2^. Twice a week the animals additionally received some fruit, vegetables or adding dead chicken and mice to their ratios of standardized feed. |
| Raccoon dog (dissemination) | 42502-3-658 IDT | Commercial fur farm,  Poland | Grouped, 2-3 animals per cage (>6m² + >20m²)^1^; 2 cages with 2 treated and one contact animal and 4 cages with 2 treated animals | 8 ♂+ 6 ♀ | adult | commercial food for fur farm animals ^2^, Twice a week the raccoon dogs additionally received some fruit, vegetables or adding dead chicken and mice to their ratios of standardized feed. |
| Raccoon dog (repeated dose) | 42502-3-658 IDT | Commercial fur farm,  Poland | Grouped, 3 animals per cage (>6m² + >20m²)^1^; 2 treated and 1 contact animal per cage | 11 ♂+ 1 ♀ | Juvenile,  (5-7 months) | commercial food for fur farm animals ^2^, Twice a week the raccoon dogs additionally received some fruit, vegetables or adding dead chicken and mice to their ratios of standardized feed. |
| Domestic cat (overdose) | 42502-3-658 IDT | Research Models and Services Harlan Laboratories, Venray, Netherlands | Grouped, 8 animals per cage (>6m² + >20m²)^1^; 6 treated and 2 contact animals per cage | 8 ♂+ 8 ♀ | Kittens,  12 – 14 weeks | Upon arrival, 23 days prior to vaccine administration, the cats received a mixture of the dog food ^3^ delivered by the supplier and dry cat food ^4^. Two days after vaccine administration, the cats were additionally offered wet cat food ^5^ for approximately one month. Afterwards, the animals received only wet and dry food for cats. |
| Domestic dog (overdose) | 42502-3-658 IDT | Research Models and Services Harlan Laboratories, Gannat, France | Grouped, 4 animals per cage (>6m² + >20m²)^1^; 3 treated and 1 contact animal per cage | 8♂+ 8♀ | puppies,  12-16 weeks | The animals received dry dog food ^3^, additionally some treats like chew bones or dried pig ears were offered. |
| Domestic dog  (dissemination) | 42502-3-762 IDT | Envigo RMS,  Gannat, France | Grouped, originally in groups of 6 animals in 2 cages (12 & 15m²) | 6 ♂+ 6♀ | Juveniles (<16 weeks) | Dry feed (“Welpenkost”) was offered as a daily ratio of 200 - 600g per animal ^5^. |
| Domestig pig (dissemination) | 42502-3-762 IDT | own breeding stock,  IDT Biologika, Dessau-Rosslau, Germany | Grouped, 2 cages with each 6 animals (>7.0m² + >20m²)^1^; one cage with 6 treated animals and the other cage with 4 treated and 2 contact animals | 12 ♂ | 6 weeks | Commercially pig feed ^6^ . |
| House mouse | 42502-3-768 IDT | Max-Planck-Institute for Evolutionary Biology, Ploen, Germany | Grouped, 10 cages (530cm²) with one treated and one naïve animal, 8 cages with one treated and two naïve animals and 2 cages with 1 treated and 3 naïve mice | 9 ♂+43♀ | Adult, 285-339 days | V1246 pellets ^7^, additionally the animals were provided carrots, apple and hay. |
| Field mouse | 42502-3-768 IDT | University of Hohenheim, Stuttgart, Germany | Grouped, 6 cages (530cm²) with 2 treated and 1 contact animal and 18 cages with one treated and one contact animal | 23♂+ 31♀ | Adults, 163 – 957 days | V1246 pellets ^7^, additionally the animals were provided carrots, apple and hay. |
| Guinea pigs | 42502-3-768 IDT | University Bielefeld, Bielefeld, Germany | Individual (>5000cm²) | 9 ♂ | Adults, approx.. 250 days | Muesli V2247 pellets ^7^, additionally the animals were provided carrots, apple and hay. |

1) - inside – and outside enclosure

2) Schirmer und Partner, Talstraße 17, 09306 Döhlen, Germany

3) Bosch Tiernahrung GmbH & Co. KG Engelhauser Str. 55+57, D-74572 Blaufelden-Wiesenbach

4) Josera GmbH &Co. KG, D-63924 Kleinheubach

5) Gerhard Vollmer GmbH & Co. KG, Industriestr. 13, D-32139 Spenge

6) Straacher Produktions- und Handels GmbH, Berkauer Str. 3, 06889 Lutherstadt Wittenberg, Germany

7) ssniff Spezial-diäten GmbH, Soest, Germany
